# Virtual triple‐bundle ACL graft via femoral tunnels behind the resident's ridge on 3D CT demonstrates equivalent orientation to native ACL

**DOI:** 10.1002/jeo2.70125

**Published:** 2025-01-03

**Authors:** Narihiro Okazaki, Konsei Shino, Hiroyuki Yokoi, Tomoki Ohori

**Affiliations:** ^1^ Sports Orthopaedic Center Yukioka Hospital Osaka Japan; ^2^ Department of Orthopaedic Surgery Osaka University Graduate School of Medicine Suita Japan

**Keywords:** ACL attachment areas, bony landmark strategy, CT‐MRI matching, normal knees, triple‐bundle ACL reconstruction

## Abstract

**Purpose:**

To clarify the femoral tunnel location for a virtual anterior cruciate ligament (ACL) graft to simulate the native ACL.

**Methods:**

Three‐dimensional (3D) computed tomography (CT) and magnetic resonance imaging (MRI) were obtained in 14 normal knees in full extension. Two types of virtual triple bundle ACL grafts (VACLG) were created. In one type, the femoral tunnels for anteromedial bundle (AM = AMM/anteromedial bundle medial part + AML/anteromedial bundle lateral part) and posterolateral bundle (PL) were positioned behind the resident's ridge (RR) based on the bone landmark strategy (BR‐VACLG group). In the other type, the tunnels were placed on the RR (OR‐VACLG group). VACLG was displayed as three straight lines by connecting the two centres of the femoral attachment areas of AM and PL to those of the three tibial footprints of AMM, AML and PL attachments on 3D CT, and then superimposed on MRI. The ACL/ACL graft‐the tibial plateau (ACL‐TP) angles were compared among normal ACL (N‐ACL), BR‐VACLG and OR‐VACLG.

**Results:**

The mean ACL‐TP angles of N‐ACL, BR‐VACLG and OR‐VACLG were 74.4 ± 3.4°, 75.2 ± 4.5° and 68.7 ± 5.0° for AMM, 81.9 ± 3.8°, 82.9 ± 5.1° and 76.3 ± 4.0° and for AML, 71.1 ± 6.4°, 70.0 ± 7.2° and 61.0 ± 4.7° for PL on the oblique‐coronal slices; 55.3 ± 4.9° 53.9 ± 4.4° and 50.5 ± 4.3° for AMM; 54.9 ± 4.5°, 54.7 ± 2.6° and 50.7 ± 3.2° for AML; 51.4 ± 3.3°, 51.2 ± 2.4° and 48.1 ± 2.0° for PL on the oblique‐sagittal slices. There was no significant difference in the angles between N‐ACL and BR‐VACLG, while those of AMM and PL in OR‐VACLG were significantly lower compared to N‐ACL.

**Conclusion:**

The virtual triple bundle ACL graft via femoral tunnels behind the RR on 3D CT shows equivalent orientation to the native ACL on MRI in full extension.

**Level of Evidence:**

Level III.

**Abbreviations**
ACL: anterior cruciate ligament
ACL‐TP angle: ACL‐tibial plateau angle
AM: anteromedial bundle
AML: anteromedial bundle lateral part
AMM: anteromedial bundle medial part
AR: anterior ridge
ATB‐ACLR: anatomical triple‐bundle ACL reconstruction
BL: bony landmark
BLS: bony landmark strategy
BR‐VACLG: virtual ACL graft via femoral tunnels behind the resident's ridge
CIR: central intercondylar ridge
F‐AM: centre of AM attachment on the femur
F‐PL: centre of PL attachment on the femur
ICC: intra‐class correlation coefficient
MIR: medial intercondylar ridge
N‐ACL: normal ACL
OR‐VACLG: virtual ACL graft via femoral tunnels on the resident's ridge
PL: posterolateral bundle
RR: resident's ridge
T‐AML: centre of AML attachment on the tibia
T‐AMM: centre of AMM attachment on the tibia
T‐PL: centre of PL attachment on the tibia
VACLG: virtual triple‐bundle ACL graft

## INTRODUCTION

The normal anterior cruciate ligament (N‐ACL) could be divided into not only two but three fibre bundles: anteromedial bundle medial part (AMM), anteromedial bundle lateral part (AML) and posterolateral bundle (PL) [8, 19]. In the anatomical triple bundle ACL reconstruction [24], it is desirable to place these three bundle grafts so that they run equivalent to those of N‐ACL. However, there is still no consensus in the tunnel position to achieve this goal, while the tunnel position affects the kinematics of the knee, and its malpositioning leads to poor clinical outcomes, including residual instability and/or graft failure [1, 3, 4, 16, 18, 31].

While the location of the tibial tunnels is almost agreeable, the position of the femoral tunnels has still been controversial [15]. Some advocated that the tunnels should be placed on the posterior part of the attachment area behind the resident's ridge (RR) [5, 9, 13, 22, 23, 24, 25], while others have been claiming those created on the resident's ridge (OR) [6, 27, 32].

In recent years, a technique for matching bone images of computed tomography (CT) and those on magnetic resonance imaging (MRI) has become available, and this has become possible to project a virtual ACL graft (VACLG) on a CT model onto MRI [7, 11, 14, 20]. This study aimed to compare the orientation of N‐ACL on MRI, BR‐VACLG/the virtual ACL graft via the femoral tunnels created behind the RR on the three‐dimensional (3D) CT and OR‐VACLG/the graft via the femoral tunnels created on the RR in the same knee. While some claim the midsubstance insertion/the resident's ridge as the tunnel location for anatomical graft placement based on macroscopic observation [6, 27, 32], other histological study suggests the area behind the ridge is more appropriate [9]. Thus, our hypothesis was that BR‐VACLG on the 3D CT showed the closer orientation to N‐ACL on MRI.

## MATERIALS AND METHODS

### Subjects

The subjects included normal left knees of healthy volunteers without a surgical or fracture history of either knee or suspected ligament instability based on manual examination or MRI. Informed consent was obtained from all the subjects involved, and the study protocol received the approval of our institution's institutional review board for human subject research.

### Scans and virtual ACLGs

**Figure 1:**
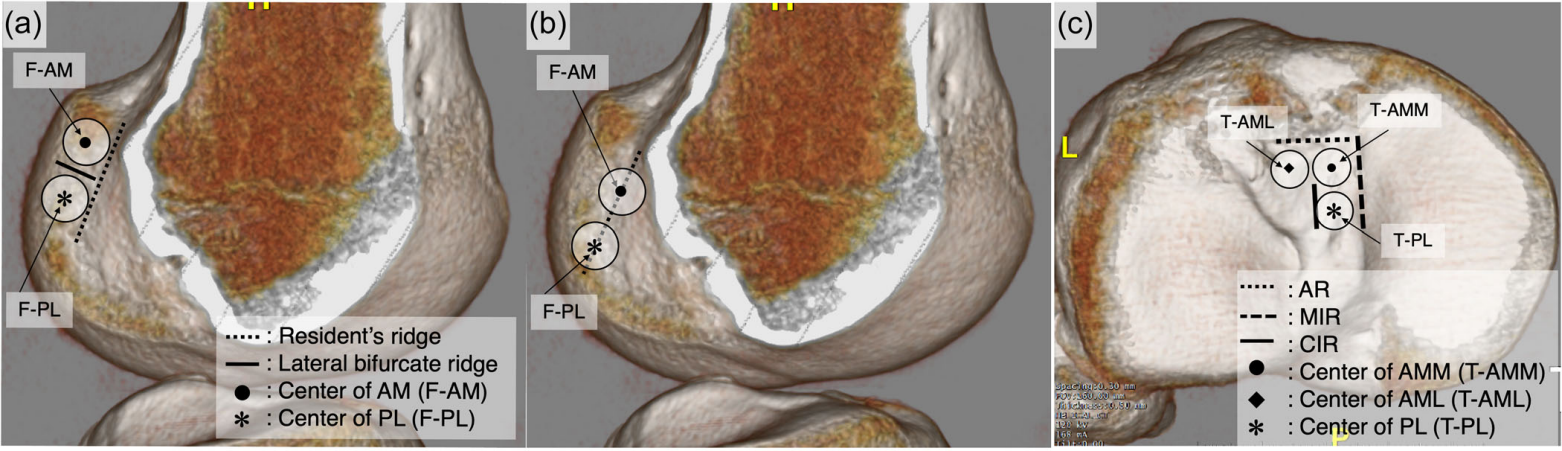
The positions of the femoral and tibial attachments of each ACL fibre bundle on 3D CT. (a) The femoral attachment site placed behind the resident's ridge (RR) (7‐mm‐diameter circle each). (b) The femoral attachment site placed on the RR (7‐mm‐diameter circle each). (c) The tibial attachment site (5‐mm‐diameter circle each). 3D, three‐dimensional; ACL, anterior cruciate ligament; AM, anteromedial bundle; AML, anteromedial bundle lateral part; AMM, anteromedial bundle medial part; AR, anterior ridge; CIR, central intercondylar ridge; F‐AM, centre of AM attachment on the femur; F‐PL, centre of PL attachment on the femur; MIR, medial intercondylar ridge; PL, posterolateral bundle; T‐AML, centre of AML attachment on the tibia; T‐AMM, centre of AMM attachment on the tibia; T‐PL, centre of PL attachment on the tibia.

**Figure 2:**
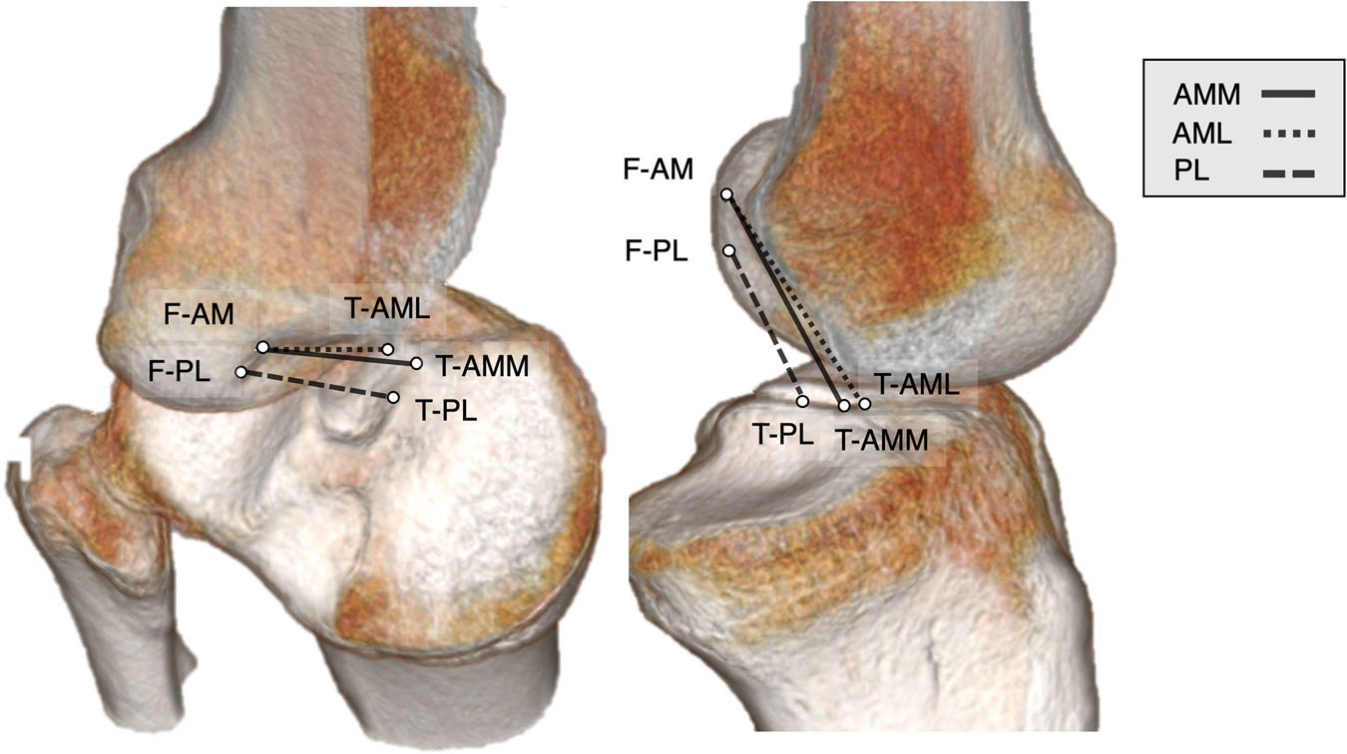
Virtual triple‐bundle ACL graft placed by connecting the centre points of each bundle's femoral and tibial attachments. ACL, anterior cruciate ligament; AM, anteromedial bundle; AML, anteromedial bundle lateral part; AMM, anteromedial bundle medial part; F‐AM, centre of AM attachment on the femur; F‐PL, centre of PL attachment on the femur; PL, posterolateral bundle; T‐AML, centre of AML attachment on the tibia; T‐AMM, point of AMM attachment on the tibia; T‐PL, centre of PL attachment on the tibia.

As virtual ACL grafts, triple‐bundle grafting was adopted to closely mimic the native ACL. On 3D CT images (Discovery CT 750HD; General Electric) with a slice thickness of 0.625 mm in the knee extension position, BLs such as the resident's ridge (RR), the lateral bifurcate ridge, the apex of the deep cartilage, Parsons’ knob (anterior ridge: AR), the medial intercondylar ridge (MIR) and the central intercondylar ridge (CIR), were identified on both the femur and tibia [5, 9, 10, 12, 13, 22, 23, 24, 25, 29, 34]. In accordance with previous reports, the area posterior to the RR on the 3D CT lateral image was defined as the femoral side attachment of the BR‐VACLG [9]. The proximal and distal parts of the lateral bifurcate ridge were defined as the AM and PL fibre attachment areas, respectively [5]. A 7‐mm‐diameter circle was created in each fibre attachment area, and their centres were defined as F‐AM and F‐PL, respectively (Figure 1a). On the tibial side, the attachment area was defined as posterior to the AR [29], lateral to the MIR, and medial to the CIR [10]. Three regular circles with a diameter of 5 mm were created in the attachments of AMM, AML and PL fibres within the attachment area, and the centres of each circle were designated T‐AMM, T‐AML and T‐PL, respectively (Figure 1c). The lines connecting the centre points of each bundle's femoral and tibial attachments were defined as VACLG (Figure 2). For comparison, the other type of VACLG: OR‐VACLG was created, in which F‐AM and F‐PL were placed on the RR, and the tibial side was placed in the same manner as the BR‐VACLG (Figure 1b).

3D MRI (EXCELART Vantage 1.5 T; Toshiba) was obtained on the same knee in the same limb position with the following protocol: T2* sequence, 512 × 512‐pixel, slice thickness 1 mm. CT‐MRI image matching system (Aquarius iNtuition viewer; TeraRecon, Inc.) was used to match the CT and MRI bones. The VACLGs were created on the MRI by projecting the centre point of each fibre bundle plotted on the CT onto the MRI.

**Figure 3:**
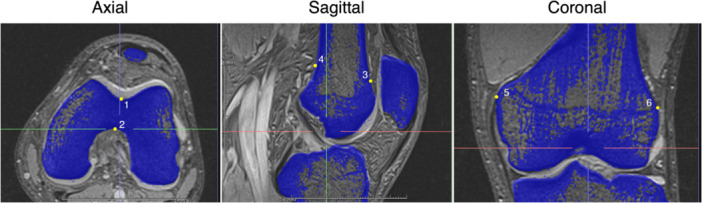
CT bone image (blue shadow) on MRI (black and white). The gaps between the CT and MR images were measured on the six bony borders: apex of the intercondylar notch (1) and bottom of the trochlea (2) on the axial slices; anterior (3) and posterior borders (4) of the femur on the sagittal slices; medial (5) and lateral borders (6) of the femur on the coronal slices. The mean gap was 0.44 ± 0.23 mm. CT, computed tomography; MRI, magnetic resonance imaging.

A validation test of CT‐MRI matching was performed with six randomly selected knees. In each knee, the gaps between the bottom point of the trochlear groove and the top of the entrance of the intercondylar notch on the axial slice, between the anterior and posterior borders of the femoral bone on the coronal slice, between the medial and lateral borders of the femoral bone on the sagittal slice were measured on both CT and MRI when matched by three orthopaedic surgeons (H.Y., R.Y. and T.H.) (Figure 3). The mean gap for 36 points (six points each in six knees) was 0.44 ± 0.23 mm, which was comparable to previous reports [7, 11, 14, 21]. Reliability calculations were performed for the bone matching between CT and MRI, and the intra‐observer and inter‐observer intra‐class correlation coefficients (ICCs) were 0.86–0.95.

### Measurements

1. ACL attachment position of each bundle of BR‐VACLG and OR‐VACLG on CT

**Figure 4:**
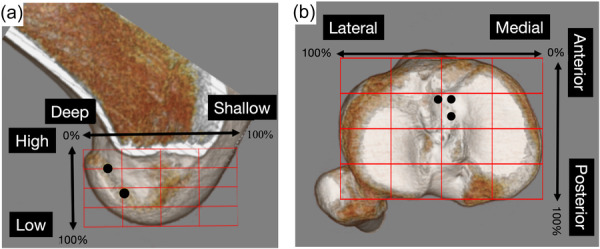
Measurements of femoral and tibial attachment positions by the quadrant method. (a) Femoral side. The two orthogonal solid red lines are perpendicular or parallel to Blumensaat's line. The posterior and superior bony edges of the lateral femoral condyles are set at 0%, and the anterior and inferior edges at 100%. (b) Tibial side. The two orthogonal solid red lines are perpendicular or parallel to the line connecting the posterior edges of the medial and lateral plateaus. The posterior and medial edges are defined as 0%, and the anterior and lateral edges as 100%.

To identify the attachment positions of F‐AM and F‐PL, the medial femoral condyle was removed so that the lateral wall of the intercondylar notch could be observed from the medial side in line with the trans‐epicondylar axis. To identify the attachment positions of T‐AMM, T‐AML and T‐PL, a bird's eye view of the tibial plateau was obtained, and a line tangential to the posterior condyles of the tibial plateau was first determined as the mediolateral axis, followed by the perpendicular anteroposterior axis. The location of the attachment position of each bundle of BR‐VACLG and OR‐VACLG was evaluated using the quadrant method on these 3D images (Figure 4a,b) [2, 30]. The intra‐observer and inter‐observer ICCs of the ACL attachment position were 0.86 and 0.78, respectively.

2. Comparison of orientation between the N‐ACL and the VACLG

**Figure 5:**
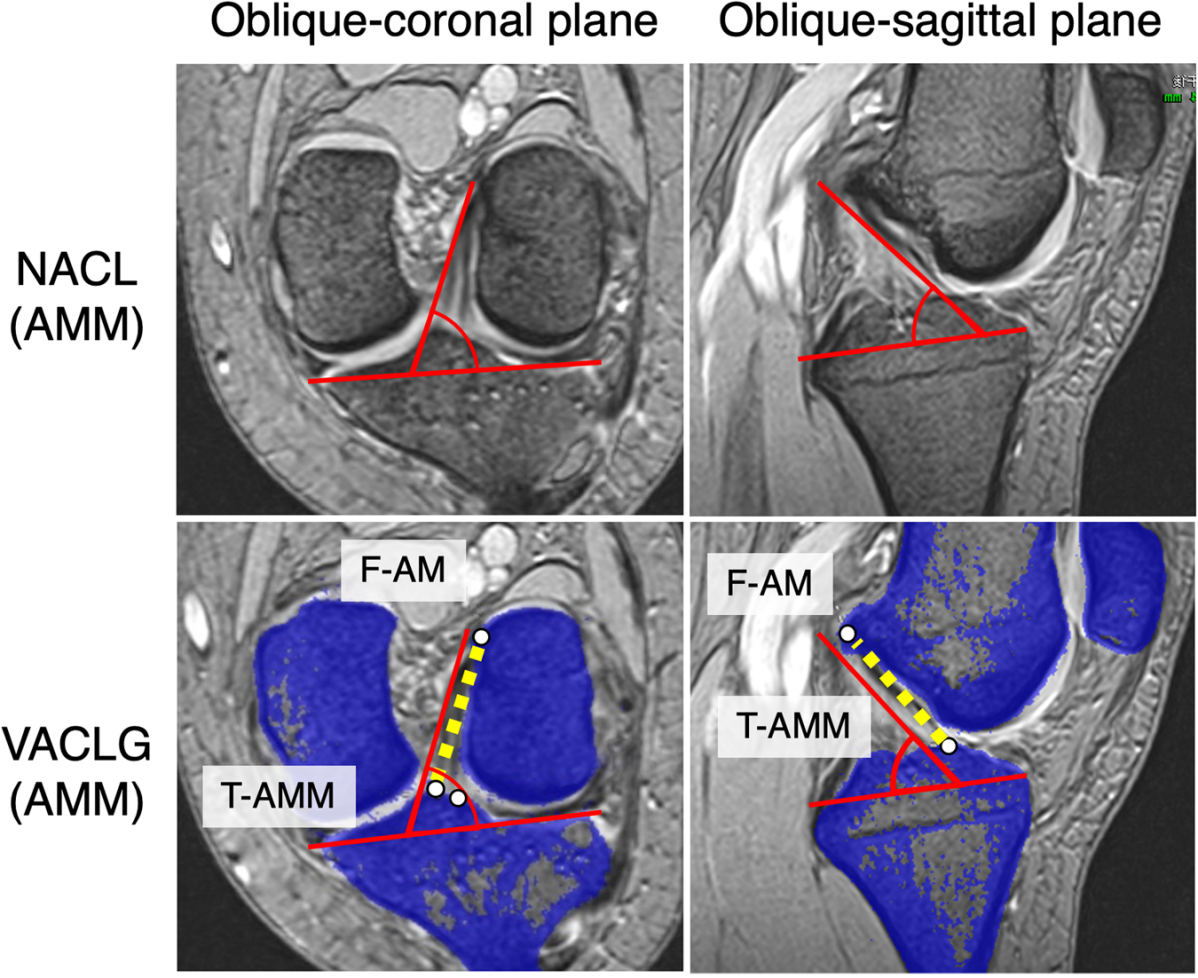
ACL‐tibial plateau angle (ACL‐TP angle) of the anteromedial bundle medial part (AMM) of the normal ACL (N‐ACL) and the virtual triple‐bundle ACL graft (VACLG) in MRI oblique‐coronal and sagittal planes. The ACL‐TP angle on the sagittal plane is the angle between the centre of the ACL (dashed line) and the approximating tibial plateau. The ACL‐TP angle in the oblique‐coronal is defined as the angle between the dashed line of the ACL and the midline of the lateral and medial tibiofemoral joint compartments. ACL, anterior cruciate ligament; MRI, magnetic resonance imaging.

The angle between the ACL bundle and the tibial articular surface (ACL‐tibial plateau angle: ACL‐TP angle) was measured in oblique‐coronal and sagittal images based on the method reported by Take et al. [28]. The tibial plateau was approximated by a plane in a scout view of frontal and lateral planes, which was defined as the axial plane. Next, the sagittal plane was defined perpendicular to the axial plane and the line tangential to the posterior edge of the tibial plateau. At the intercondylar level of sagittal slices, ACL grafts were clearly visible running obliquely beneath the intercondylar roof, or Blumensaat's line. The depicted ACL graft in the sagittal plane was then used to determine the oblique‐coronal plane, which contained a line tracing the centre of the ACL graft, and it was also set parallel to the line tangent to the posterior edge of the tibial plateau. The 3D orientation of the ACL bundle was represented by the ACL‐TP angle measured in the oblique‐coronal and sagittal planes. The ACL‐TP angles of the BR‐VACLG and OR‐VACLG were measured with the same slices used for the N‐ACL measurements. ACL‐TP angles of each bundle in the BR‐VACLG, OR‐VACLG, and N‐ACL were compared (Figure 5). The intra‐observer and inter‐observer ICCs of ACL‐TP angle measurements were 0.81 and 0.65, respectively.

### Statistical analysis

Statistical analysis was performed using SPSS Statistics ver. 26 (IBM Corp.). The femoral attachment positions of each bundle of BR‐VACLG and OR‐VACLG by the Quadrant method were compared using the Wilcoxon signed‐rank test, with an alpha level of 0.05. Comparisons of the ACL‐TP angle of each bundle among N‐ACL, BR‐VACLG and OR‐VACLG were performed using the Friedman test with Bonferroni adjustment (*α* = 0.05/3 = 0.0167).

## RESULTS

A total of 14 normal left knees (7 males and 7 females, mean age 27.8 ± 4.6 years) were included.

1. Tunnel positions of each bundle based on the BLS

On BR‐VACLG, the femoral tunnels were located at 13.6 ± 2.5%/22.1 ± 9.0% for the F‐AM and 25.3 ± 3.1%/52.4 ± 7.0% for the F‐PL in deep‐shallow/high‐low directions by the quadrant coordinate system (Table 1). The femoral tunnels of OR‐VACLG were located at 24.9 ± 2.8%/30.5 ± 3.6% for the F‐AM and 37.2 ± 4.2%/59.3 ± 3.9% for the F‐PL in deep‐shallow/high‐low directions. Hence the femoral tunnels of the OR‐VACLG were statistically significantly shallower and lower compared to those of the BR‐VACLG in both F‐AM (*p* = 0.012 and *p* = 0.036, respectively) and F‐PL (*p* = 0.012 and *p* = 0.018, respectively).

**Table 1:** Femoral tunnel location by the quadrant method.

	Deep‐shallow			High‐low		
	BR‐VACLG	OR‐VACLG	*p*	BR‐VACLG	OR‐VACLG	*p*
AM	13.6 ± 2.5%	24.9 ± 2.8%	**0.012**	22.1 ± 9.0%	30.5 ± 3.6%	**0.036**
PL	25.3 ± 3.1%	37.2 ± 4.2%	**0.012**	52.0 ± 7.0%	59.3 ± 3.9%	**0.018**

*Note*: Data are presented as the mean ± standard deviation. The deepest or the highest border was defined as 0%. Wilcoxon signed‐rank test: bold text indicates statistical significance.

Abbreviations: ACL, anterior cruciate ligament; AM, anteromedial bundle; BR‐VACLG, virtual triple‐bundle ACL graft via femoral tunnels behind the resident's ridge; OR‐VACLG, virtual triple‐bundle ACL graft via femoral tunnels on the resident's ridge; PL, posterolateral bundle.

The tibial tunnels were located at 44.0 ± 1.5%/23.4 ± 2.9% for the T‐AMM, 50.0 ± 1.9%/28.0 ± 2.9% for the T‐AML and 43.5 ± 1.6%/39.2 ± 3.0% for the T‐PL in medial‐lateral/anterior‐posterior directions (Table 2).

**Table 2:** Tibial tunnel location by the quadrant method.

	Mediolateral	Anteroposterior
AMM	44.0 ± 1.5%	23.4 ± 2.9%
AML	50.0 ± 1.9%	28.0 ± 2.9%
PL	43.5 ± 1.6%	39.2 ± 3.0%

*Note*: Data are presented as the mean ± standard deviation. Medial or anterior border was defined as 0%.

Abbreviations: AML, lateral portion of the anteromedial bundle; AMM, medial portion of the anteromedial bundle; PL, posterolateral bundle.

2. Comparison in orientation among N‐ACL, BR‐VACLG and OR‐VACLG

**Figure 6:**
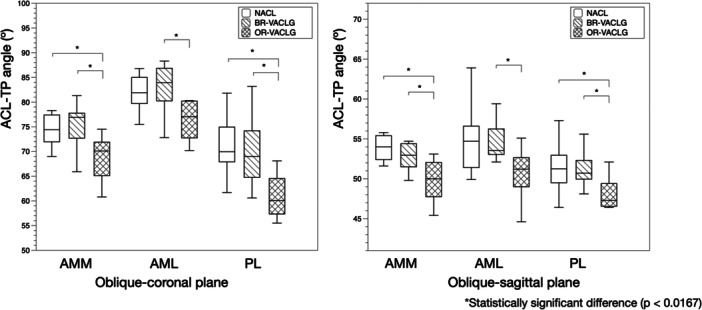
The ACL‐tibial plateau angle (ACL‐TP angle) of the normal ACL (N‐ACL), virtual ACL via femoral tunnels behind the resident's ridge (BR‐VACLG), and the virtual ACL via femoral tunnels on the resident's ridge (OR‐VACLG) in the oblique‐coronal plane and sagittal plane. There is no significant difference for either fibre bundle of N‐ACL and BR‐VACLG in both planes. In contrast, the ACL‐TP angles of AMM and PL in OR‐VACLG are significantly lower than those of N‐ACL in both planes, and those of OR‐VACLG are significantly lower than those of BR‐VACLG for all three bundles in both planes. ACL, anterior cruciate ligament; AML, anteromedial bundle lateral part; AMM, anteromedial bundle medial part; PL, posterolateral bundle.

**Figure 7:**
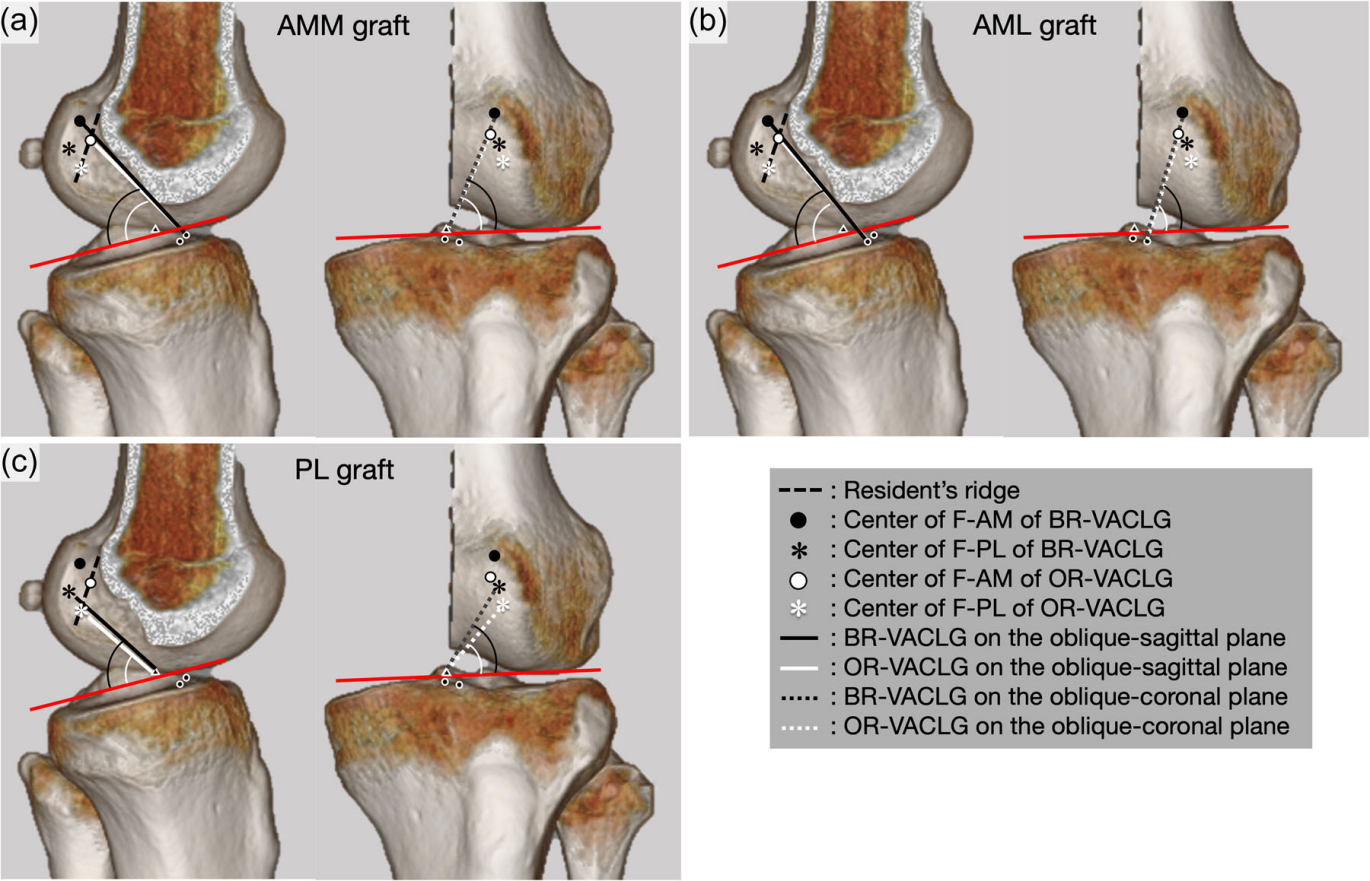
The difference in the ACL‐tibial plateau angle (ACL‐TP angle) between the virtual ACL via femoral tunnels behind the resident's ridge (BR‐VACLG) and the virtual ACL via femoral tunnels on the resident's ridge (OR‐VACLG). (a) AMM, anteromedial bundle medial part. (b) AML, anteromedial bundle lateral part. (c) PL, posterolateral bundle. The ACL‐TP angle of OR‐VACLG is lower than that of BR‐VACLG in the oblique‐coronal image. Red line: tibial plateau. ACL, anterior cruciate ligament; F‐AM, centre of AM attachment on the femur; F‐PL, centre of PL attachment on the femur.

ACL‐TP angles of the BR‐VACLG and N‐ACL were 75.2 ± 4.5° and 74.4 ± 3.4° for AMM, 82.9 ± 5.1° and 81.9 ± 3.8° for AML, 70.0 ± 7.2° and 71.1 ± 6.4° for PL in oblique‐coronal slices; 53.9 ± 4.4°and 55.3 ± 4.9° for AMM; 54.7 ± 2.6° and 54.9 ± 4.5° for AML; 51.2 ± 2.4° and 51.4 ± 3.3° for PL in oblique‐sagittal slices. There was no significant difference in the angles between N‐ACL and BR‐VACLG on the oblique‐coronal slices (*p* = 0.617 for AMM, p = 0.261 for AML, and *p* = 0.617 for PL) or the oblique‐sagittal slices (*p* = 0.211 for AMM, *p* = 0.453 for AML, and *p* = 0.901 for PL) (Table 3, Figures 6 and 7).

**Table 3:** ACL‐tibial plateau angle (°).

		N‐ACL (N)	BR‐VACLG (B)	OR‐VACLG (O)	*p*		
					N vs. B	N vs. O	B vs. O
Oblique‐ coronal plane	AMM	74.4 ± 3.4	75.2 ± 4.8	68.7 ± 5.0	0.617	**0.006**	**0.001**
	AML	81.9 ± 3.8	82.9 ± 5.1	76.3 ± 4.0	0.261	0.024	**<0.001**
	PL	71.1 ± 6.4	70.0 ± 7.2	61.0 ± 4.7	0.617	**0.001**	**0.006**
Oblique‐sagittal plane	AMM	55.3 ± 4.9	53.9 ± 4.4	50.5 ± 4.3	0.211	**<0.001**	**0.018**
	AML	54.9 ± 4.5	54.7 ± 2.6	50.7 ± 3.2	0.453	0.024	**0.003**
	PL	51.4 ± 3.3	51.2 ± 2.4	48.1 ± 2.0	0.901	**0.012**	**0.018**

*Note*: Data are presented as the mean ± standard deviation. Friedman test with Bonferroni adjustment (*α* = 0.05/3 = 0.0167): Bold text indicates statistical significance.

Abbreviations: ACL, anterior cruciate ligament; AML, lateral portion of the anteromedial bundle; AMM, medial portion of the anteromedial bundle; BR‐VACLG, virtual triple‐bundle ACL graft via femoral tunnels behind the resident's ridge; N‐ACL, normal ACL; OR‐VACLG, virtual triple‐bundle ACL graft via femoral tunnels on the resident's ridge; PL, posterolateral bundle.

The angles of the OR‐VACLG were 68.7 ± 5.0° for AMM, 76.3 ± 4.0° for AML, and 61.0 ± 4.7° for PL in the oblique‐coronal slice, and 50.5 ± 4.3° for AMM, 50.7 ± 3.2° for AML, and 48.1 ± 2.0° for PL in the oblique‐sagittal slices (Table 3). The angles of AMM and PL in OR‐VACLG were statistically significantly lower than those of N‐ACL on the oblique‐coronal slices (*p* = 0.006 for AMM and *p* = 0.001 for PL) or the oblique‐sagittal slices (*p* < 0.001 for AMM and *p* = 0.012 for PL), and those for all three bundles of OR‐VACLG were statistically significantly lower than those of BR‐VACLG on the oblique‐coronal slices (p = 0.001 for AMM, *p* < 0.001 for AML, and *p* = 0.006 for PL) or the oblique‐sagittal slices (*p* = 0.018 for AMM, *p* = 0.003 for AML, and *p* = 0.018 for PL) (Table 3, Figures 6 and 7).

## DISCUSSION

The most important finding of this study was that the BR‐VACLG showed a similar orientation to the N‐ACL on MRI in the normal fully extended knees. Comparison in ACL‐TP angles of each fibre bundle on both oblique‐coronal and sagittal slices between the BR‐VACLG on the CT model and the N‐ACL on MRI, showed no significant differences. In other words, each fibre bundle in the BR‐VACLG based on the BLS accurately mimicked the orientation of the N‐ACL, in contrast to that in the OR**‐**VACLG.

In anatomic ACL reconstructions, it could reasonably be assumed to mimic the normal ACL orientation by creating tunnels within the ACL attachment areas that have some BLs in their vicinity, as reported in the past. Iwahashi et al. reported that the ACL femoral attachment area was attached to the concave region behind the RR by histological evaluation [9]. Ferretti et al. reported the presence of the lateral bifurcate ridge between the AM and PL bundles in the femoral attachment area [5]. The ACL tibial attachment has been reported to have a C‐ or boot‐like shape [26, 33]. Tensho et al. reported that the ACL is attached to the posterior medial side of the bony prominence of the L‐shape formed by the bony landmarks AR and MIR [29]. Kusano et al. reported that the CIR is located at the boundary between the ACL and the anterior horn of the lateral meniscus [10]. The size of the attachment areas is reported as follows: 15–18 mm in length and 7–10 mm in width on the femoral side; 7–11 mm in length for AR, 10–13 mm in length for MIR, about 5 mm in length for CIR and about 5 mm of MIR‐CIR distance on the tibial side [10, 13, 20, 22, 29]. Thus, in the present study, based on the BLS, two 7‐mm‐diameter circles straddling the lateral bifurcate ridge in the area behind the RR were used as the ACL femoral attachment points for the AM (AMM + AML) and PL bundles, respectively. Three circles, 5 mm in diameter, placed posterior to the AR, lateral to the MIR and medial to the CIR, were used as ACL tibial attachment points for the AMM, AML and PL bundles, respectively. Then, VACLG of the three lines connecting the centres of each fibre bundle could closely mimic the N‐ACL in the extended knee, showing that anatomic grafting in ACL reconstruction could be achieved by the BLS.

Clinically, Take et al. evaluated the tunnel position of ATB‐ACLR based on the BLS by the quadrant method. F‐AM was 13.2%: 21.1%, F‐PL 20.0%: 48.5% (deep‐shallow: high‐low directions) on the femoral side, while T‐AMM was 44.1%: 26.8%, T‐AML was 49.6%: 27.3% and T‐PL was 45.4%: 38.0% (medial‐lateral: anterior‐posterior directions) on the tibial side, which were comparable to the tunnel positions for BR‐VACLG in the present study [28]. They also reported excellent clinical results in which the mean side‐to‐side difference of anterior laxity measured by the KT‐1000 arthrometer at one year after ATB‐ACLR was 0.4 ± 1.2 mm with the tibio‐femoral positional relationship normalised [17, 28]. Thus, the BLS could make it possible to reproducibly create anatomic tunnels leading to consistent restoration of stability, as proposed by Shino et al. [25].

The present study has some limitations. First, the sample size is small, so statistical power is reduced. Second, there were some cases in which the BLs were not clearly depicted on CT. It has been reported that 100% of the RR [20], 96%–100% of the AR [29, 33] and 100% of the MIR and CIR can be visualised on CT [33], but only about 80% of the lateral bifurcate ridge can be visualised by histological and arthroscopic examination [5]. In the present study, the RR, AR, MIR and CIR were 100% delineable, but the lateral bifurcate ridge was unclear in 4 out of 26 knees (16%). However, the two 7‐mm‐diameter regular circles placed posterior to the RR in this study were considered properly positioned within the femoral attachment of the ACL, and the results would be similar even if the lateral bifurcate ridge were unclear. Third, the ACL‐TP angle was evaluated in a non‐weight‐bearing, static position. The CT and MRI images of the same knee had to be completely overlapped, so the scans were performed in extension under non‐weight‐bearing conditions. However, the conclusion drawn from this study may be different in the other conditions, as ACL tension/length is influenced by the knee flexion angle, weight‐bearing and quadriceps muscle contraction.

## CONCLUSION

The virtual triple‐bundle ACL graft on the 3D CT via the femoral tunnels behind the resident's ridge showed the equivalent orientation to the normal ACL on MRI.

## AUTHOR CONTRIBUTIONS

Konsei Shino and Hiroyuki Yokoi conceived the hypothesis and designed this study. Hiroyuki Yokoi, Tomoki Ohori and Konsei Shino collected data. Narihiro Okazaki, Konsei Shino and Hiroyuki Yokoi analysed and interpreted the results and drafted the manuscript. Tomoki Ohori supported statistical analyses. All authors read and approved the final manuscript.

## CONFLICT OF INTEREST STATEMENT

The authors declare no conflicts of interest.

## ETHICS STATEMENT

The research related to human use has been conducted in accordance with all the relevant national regulations and institutional policies and the tenets of the Helsinki Declaration, and it has been approved by the institutional review board of Yukioka Hospital. Informed consent has been obtained from all individuals included in this study.

## Data Availability

The data that support the findings of this study are available from the corresponding author upon reasonable request.
